# Effects of virtual reality-based intervention on depression in stroke patients: a meta-analysis

**DOI:** 10.1038/s41598-023-31477-z

**Published:** 2023-03-16

**Authors:** Hebing Liu, Zhaohua Cheng, Shuo Wang, Yong Jia

**Affiliations:** 1 The Second Hospital, Jilin University, No. 218 Ziqiang Street, Changchun, 130021 Jilin China; 2 School of Nursing, Xuzhou Medical University, Tongshan Road, Xuzhou, 221004 Jiangsu China; 3 School of Nursing, Jilin University, No.965 Xinjiang Street, Changchun, 130021 Jilin China

**Keywords:** Diseases, Neurology

## Abstract

As one of the most common neuropsychiatric complications after stroke, post-stroke depression can significantly affect the initiative of rehabilitation exercise and the rehabilitation of neurological function of patients. Virtual reality (VR) has been widely used in health-related fields in recent years. There is some evidence that VR-based interventions have benefits for depression. The aim of this study was to assess the effectiveness of VR-based intervention on depression in stroke patients. A total of 752 patients with stroke from 11 randomized controlled trials (RCTs) studies were included in this meta-analysis and the studies derived from seven electronic databases searched from database inception to August 2021. Different tools were used to measure depression. For continuous results, the standardized mean differences (SMDs) and 95% confidence intervals (CIs) were calculated to synthesize the effects. We assessed the risk of bias by using the Cochrane Collaboration criteria. The results showed that compared to the control group, VR-based interventions significantly decreased the depression scale score (SMD =  − 0.75, 95% CI − 1.35, − 0.15). The meta-analysis indicated that VR-based intervention had a moderate effect on depression in stroke patients compared to control group. There was no evidence of potential publication bias as assessed by visual inspection of funnel plots in Egger and Begg tests. Substantial heterogeneity between studies was observed, meta-regression analysis showed that mean age might be the source of heterogeneity.

## Introduction

Stroke, as one of the most common neurological diseases resulting in permanent disability in adults^1^, has become a widespread health problem that affects people’s daily independent living^2–4^. For patients with stroke, the risk of impaired psychological health and depression increases^5^. About a third of stroke survivors experience post-stroke depression^6^. Patients with depression can lead to loss of interest and happiness, and produce anxiety, fear, hostility, sadness and anger^7^. For stroke patients, post-stroke depression not only delays the recovery of neurological deficits but also harms functional recovery and rehabilitation^8–11^, resulting in a decline in health-related quality of life^12^ and increasing mortality^8^. Such psychological states may lead to reduced motivation and low adherence to rehabilitation training^5^.

The current intervention methods mainly include drugs and psychotherapy. Studies have shown that up to 43% of patients with major depressive disorder may discontinue antidepressants due to adverse reactions during treatment^13^. So non-drug interventions are getting more and more attention. In recent years, VR-based intervention has been found to be effective for depression^14–16^. VR is a computer-generated high-tech simulation system that can create a sense of being there using computer electronic information simulation technology^17^. The characteristics of VR include non-immersion, immersion, interaction, and imagination^18–21^ etc. VR training, as a dynamic training method in line with daily life activities, can carry out active rehabilitation training from three levels of repetition, feedback, and motivation in a computer simulation of three-dimensional space. Therefore, this method can increase patients’ subjective initiative through multi-sensory feedback.

VR is widely used to treat people such as stroke^21–23^, chronic kidney^24^, and cerebral palsy^25^ to improve mobility, balance, or walking speed. The interventions based on VR are diverse, including virtual reality exposure therapy^26,27^, virtual reality cognitive behavioral therapy^28,29^, virtual reality sports games^30^, etc. Compared with traditional therapy, VR is more convenient to use, flexible and changeable in treatment content, more positive in users’ attitudes, and more pleasant in experience^31^. A variety of games can enable patients to have a deeper interest, and thus improve their subjective initiative resulting in various rehabilitation training being actively completed by patients^32^. As a result, a virtuous cycle can be formed, and their functional level is improved^33^.

Compared to traditional conventional rehabilitation training, VR is thought to reduce learning difficulty and make training safer^34^. However, the application of VR technology in the treatment of depression has not been promoted in clinical practice, and its effect is still being explored. While two^22,23^ meta-analyses have explored VR intervention for depression, they did not specify focus on the stroke population. Thus, the purpose of this study was to specifically analyze the effects of virtual reality-based intervention on depression in stroke patients.

## Methods

Systematic review and meta-analysis were conducted in accordance with the Preferred Reporting Items for Systematic Reviews and Meta-Analyses (PRISMA) guidelines^35^. A study protocol was registered at PROSPERO (CRD42020194244).

### Literature inclusion and eligibility criteria

Studies were selected based on the following criteria: (a) study design must be randomized controlled trial (RCT); (b) the participants are stroke patients and older than 18 years old; (c) interventions in the experiment group were included all kinds of VR intervention, including VR games with exercise or VR psychotherapy. There was no restriction on the immersive levels of the VR environment displayed by the devices, such as a television, a screen, or a head-mounted display; (d) interventions in the control group are conventional rehabilitation training without VR, and not combined with other training. If there are both an active treatment group and a waiting-list group in the control group, the waiting-list group is selected; (e) depression is the primary outcome, and it must be measured. Useful data (e.g., sample size, mean and standard deviation) were reported in the published paper or could be retrieved by contacting the corresponding author of primary studies; (f) full-text articles published in peer-reviewed journals; (g) the number of participants including the experiment group and the control group is over 10; (h) the articles are published in Chinese or English.

### Data sources and search strategy

Comprehensive systematic literature was performed using four English databases and three Chinese databases: Medline, PubMed, Web of Science, CINAHL, Chinese National Knowledge Infrastructure (CNKI), SinoMed (CBM), and Wanfang databases. Seven databases were searched from construction until August 2021. The following search items were adopted: virtual reality, VR, virtual environment, depression, depressive symptoms, depressive disorder, stroke, randomized controlled trial. Search strategies were included in Supplementary Appendix 1.

### Study selection

Duplicate papers would be removed first before undertaking the screening. Two reviewers (HBL and ZHC) checked independently titles and abstracts of the retrieved articles according to the review criteria. Any disagreement was resolved by consensus and a third reviewer (SW) would arbitrate disagreements.

### Data extraction

An electronic form was established to extract substantial contents, which includes the first author and publish year of the study, the sample size, nation, age, condition, pre-treatment and post-treatment score for depression, intervention period and frequency, the assessment tools for outcome measures, and interventions in experimental group and control group. Data extraction was carried out independently by two authors (HBL and ZHC). Any disagreement was be resolved by consensus and a third reviewer (YJ) was arbitrate disagreements.

### Quality assessment

Based on the Cochrane Collaboration Risk of Bias tool^36^, the assessment of the methodological quality of included studies was assessed respectively independently by two review authors (HBL and ZHC) to evaluate the potential bias risk. Six perspectives pertained to bias risk including random sequence generation, allocation concealment, blinding of participants and personnel, blinding of outcome assessment, selective reporting, and other issues were also evaluated. Using this tool, the risk of bias was judged as ‘Low’, ‘High’, or ‘Unclear’.

### Data analysis

Software stata 16.0 was used to analyze the outcome indicators of the included studies. The tools that measured depression were not always the same. For continuous results, the standardized mean difference (SMD) and 95% confidence intervals (CIs) were calculated. *I*^2^ was used to evaluate the heterogeneity in this study. When *I*^2^ ≤ 24%, there is no heterogeneity; 25% ≤ *I*^2^ ≤ 49%, low heterogeneity; 50% ≤ *I*^2^ ≤ 74%, mild heterogeneity; *I*^2^ ≥ 75%, high heterogeneity^36^. When I^2^ > 50%, the substantial heterogeneity was considered and a random effect model was selected, otherwise, a fixed effect model was selected for analysis^33^. Publication bias was explored using a funnel plot, Egger test^37^ and Begg test^38^. Sensitivity tests were also conducted to ensure the stability of the meta-analysis. Subgroups analyses explore potential differences and compare effect sizes between VR groups and control groups. Meta-regression was performed to explore the source of heterogeneity.

## Results

### Study selection

**Figure 1:**
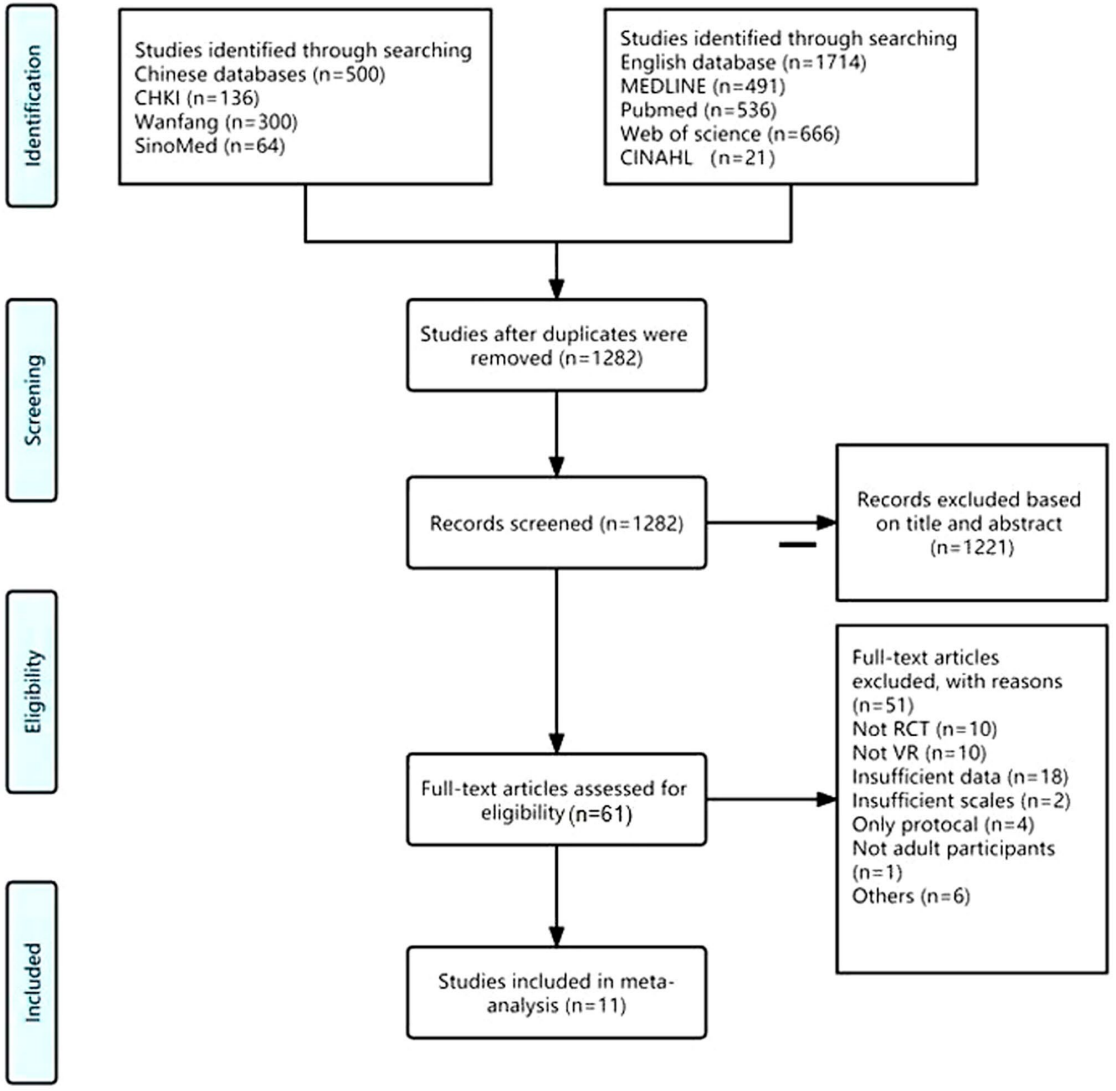
Flow chart of the study selection process in meta-analysis.

Figure 1 shows the study selection process. In total, 2214 records were identified by searching the databases, and 1282 potentially eligible articles were identified after removing duplicates. 61 studies were retrieved and reviewed by a more detailed assessment after screening the titles and abstract. After reviewing the full-text articles for eligibility, 11 studies were included.

### Characteristics of included studi*es*

Table 1 shows the characteristics of the included studies, in which the characteristics of experimental and control groups were shown in Supplementary Appendix 2. In this study, the 11 RCTs included were published between 2015 and 2021, including six English studies and five Chinese studies. The population sizes of the trials ranged from 21 to 145. All trials are recruited participants from the hospital who have been diagnosed with different types of stroke. Participants in all trials were not prescribed antidepressants. Among them, only one trial^39^ employed samples have been diagnosed with depression. The session duration of each intervention ranged from 15 to 60 min. One trial^40^ is a 2 weeks intervention period, five trials^5,31,41–43^ are a 4 weeks intervention period, two trials^44,45^ are a 6 weeks intervention period, and three trials^39,46,47^ are an 8 weeks intervention period. Six of the studies^5,31,40,42,44,45^ involved people with mild depression, five^39,41,43,46,47^ with moderate depression, and none with severe depression.

**Table 1:** Characteristics of the studies included the meta-analysis.

Author (year)	Country Region	Sample size/female	Age	Condition	Pre-treatment score for depression	Post-treatment score for depression	Intervention period and frequency	Depression measures
Adomaviciene et al. (2019)^38^	Lithuania Vilnius	42/14	EG: 62 ± 5.9	Stroke	EG: 8.40 ± 4.44	EG: 8.48 ± 4.43	45 min/time, 1time/day, 5 times/week, 2 weeks	HADS-D
			CG: 66 ± 7.0		CG: 5.41 ± 3.12	CG: 4.94 ± 3.09		
Bi et al. (2020)^37^	China Ningbo	120/52	EG: 68.3 ± 8.3	Post stroke depression	EG: 70.37 ± 10.13	EG: 46.33 ± 7.42	40–60 min/time, 1 time/day, 3–4 times/week, 8 weeks	SDS
			CG: 67.9 ± 6.1		CG: 68.13 ± 10.83	CG: 56.73 ± 4.81		
Kim et al. (2020)^39^	Korea Seoul	24/17	EG: 71.92 ± 3.23	Stroke	EG: 16.00 ± 1.04	EG: 12.16 ± 0.71	30 min/time, 1 time/day, 3 times/week, 4 weeks	K-GDS
			CG: 72.08 ± 4.46		CG: 17.08 ± 1.37	CG: 12.75 ± 0.35		
Lin et al. (2020)^5^	China Taiwan	145/87	EG: 64.5 ± 13.5	Stroke	EG: 12.1 ± 2.5	EG: 9.3 ± 3.2	15 min/time, 2 time/day, 5 times/week, 4 weeks	HADS-D
			CG: 66.9 ± 13.3		CG: 10.3 ± 4.8	CG: 10 ± 4.5		
Rogers et al. (2019)^29^	Australia Sydney	21/12	EG: 64.3 ± 17.4	Sub-acute stroke	EG: 32.6 ± 9.1	EG: 24.5 ± 6.6	30–40 min/time, 1 time/day, 3 times/week, 4 weeks	NFI Sub-scale
			CG: 64.6 ± 12.0		CG: 33.9 ± 13.9	CG: 30.6 ± 10.6		
Rooij et al. (2021)^42^	Netherland Breda	55/16	EG: 65 ± 9.6	Stroke	EG: 4.39 ± 3.35	EG: 4.04 ± 3.49	30 min/time, 1 time/day, 2 times/week, 6 weeks	HADS-D
			CG: 61 ± 13.3		CG: 3.54 ± 2.28	CG: 2.83 ± 2.16		
Song et al. (2015)^45^	Korea Daegu	40/18	EG: 51.37 ± 40.6	Stroke	EG: 21.2 ± 3.8	EG: 14.1 ± 2.4	30 min/time, 1time/day, 5 times/week, 8 weeks	BDI
			CG: 50.10 ± 7.83		CG: 19.6 ± 3.2	CG: 17.5 ± 2.7		
Sun et al. (2018)^44^	China Zhejiang	64/23	EG: 50.5 ± 9.6	Stroke	EG: 21.8 ± 3.4	EG: 8.7 ± 2.8	15 min/time, 1time/day, 6 times/week, 8 weeks	HAMD
			CG: 53.0 ± 8.7		CG: 20.8 ± 3.2	CG: 12.3 ± 3		
Xu et al. (2020)^41^	China Zhengzhou	72/26	EG: 52.33 ± 5.37	Post stroke tilting syndrome	EG: 15.56 ± 3.11	EG: 10.17 ± 2.01	50 min/time, 1time/day, 6 times/week, 4 weeks	HAMD
			CG: 51.67 ± 4.61		CG: 16.72 ± 3.23	CG: 14.26 ± 2.95		
Yu et al. (2020)^41^	China Hebei	109/41	EG: 39.56 ± 4.83	Ischemic stroke	EG: 28.15 ± 1.23	EG: 11.25 ± 2.12	20–30 min/time, 2 times/day, 7 times/week, 4 weeks	HAMD
			CG: 40.24 ± 4.83		CG: 29.23 ± 2.24	CG: 16.24 ± 1.64		
Zhang et al. (2017)^43^	China Wengzhou	60/18	EG: 67.80 ± 5.76	Stroke hemiplegia	EG: 8.83 ± 3.09	EG: 7.57 ± 2.14	30 min/time, 1time/day, 5 times/week, 6 weeks	HADS-D

*EG* experimental group, *CG* control group, *HADS-D* hospital anxiety and depression scale for depression, *SDS* self-rating depression scale, *HAMD* hamilton depression scale, *BDI* beck depression inventory, *K-GDS* Korea geriatric depression scale, *VRGs* virtual reality games, *VRT* virtual reality gait training, *NFI* Neurobehavioral Functioning Inventory.

Assessment tools for depression included Hospital Anxiety and Depression Scale for Depression, Beck Depression Inventory, Hamilton Depression Scale, Self-rating depression scale, Korea geriatric depression scale, and Neurobehavioral Functioning Inventory Sub-scale.

### Risk of bias

**Figure 2:**
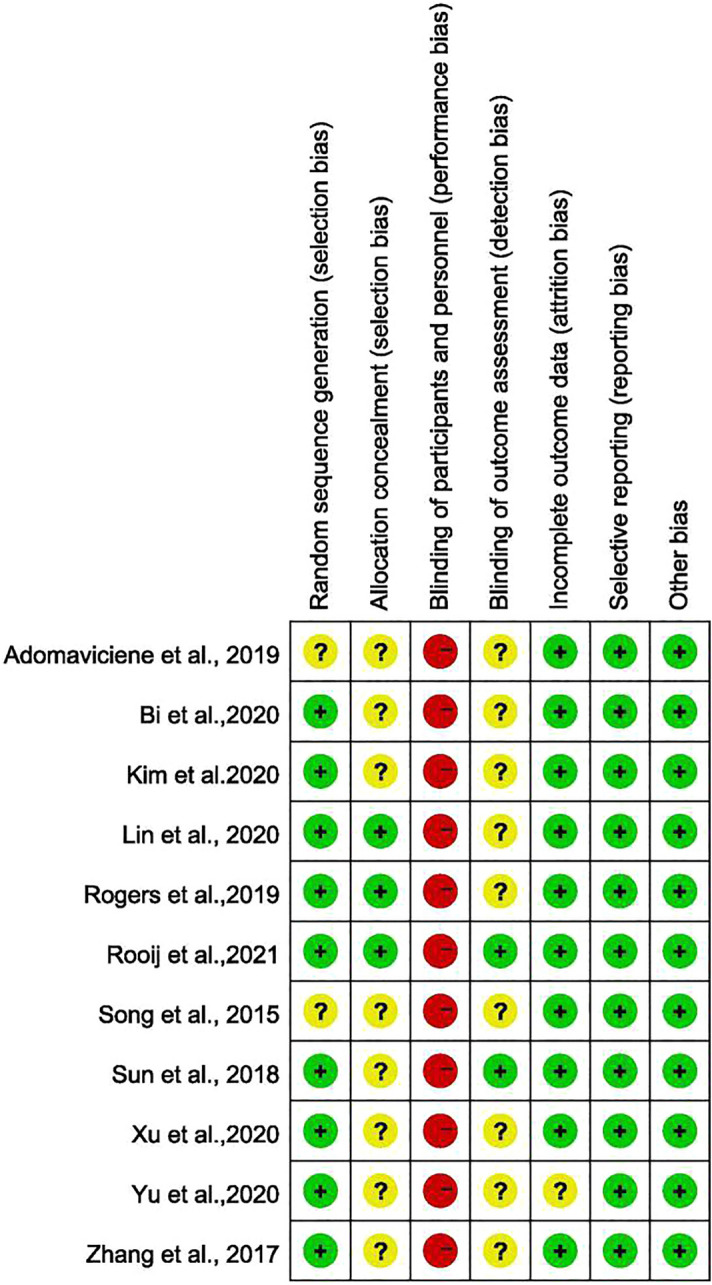
Summary of risk-of-bias distribution across studies.

The risk-of-bias results are summarized for the included studies in Fig. 2. All the studies were described as randomized. Nine studies^5,12,24,41–46^ described the method of randomization used. Information on concealment of treatment allocation was reported in three studies^5,31,44^. No study described clearly blinding of the participants and personnel. VR interventions are difficult to blind, so blindness of participants and personnel was assessed as having a high risk of bias in all studies. Two trials^44,46^ reported adequate blinding of the outcome assessor. There was no study at unclear risk of bias about incomplete outcome data. The risk of bias due to selection reporting was low across all the studies. No other potential bias was found in all studies.

### The effect of VR-based interventions on depression

**Figure 3:**
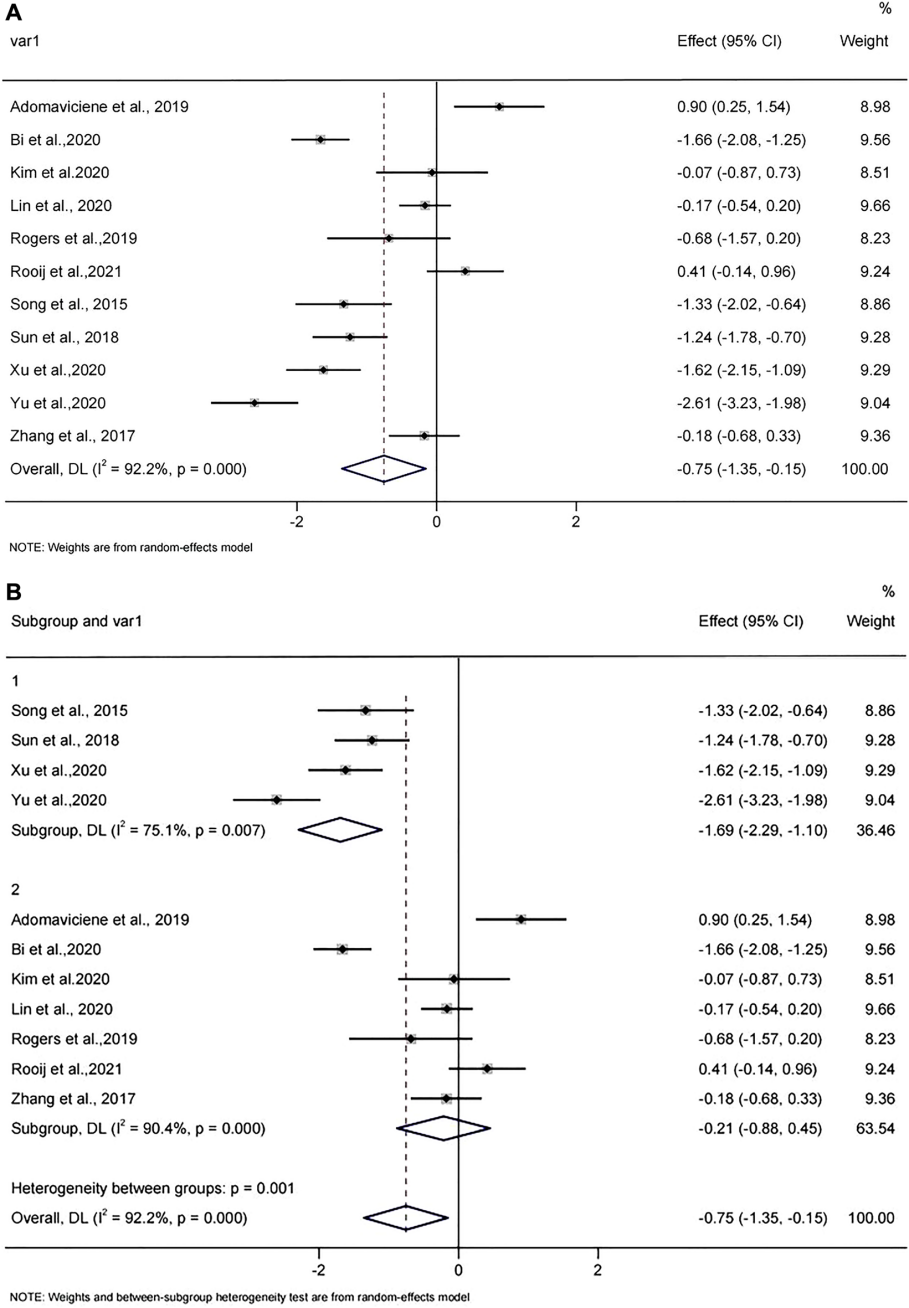

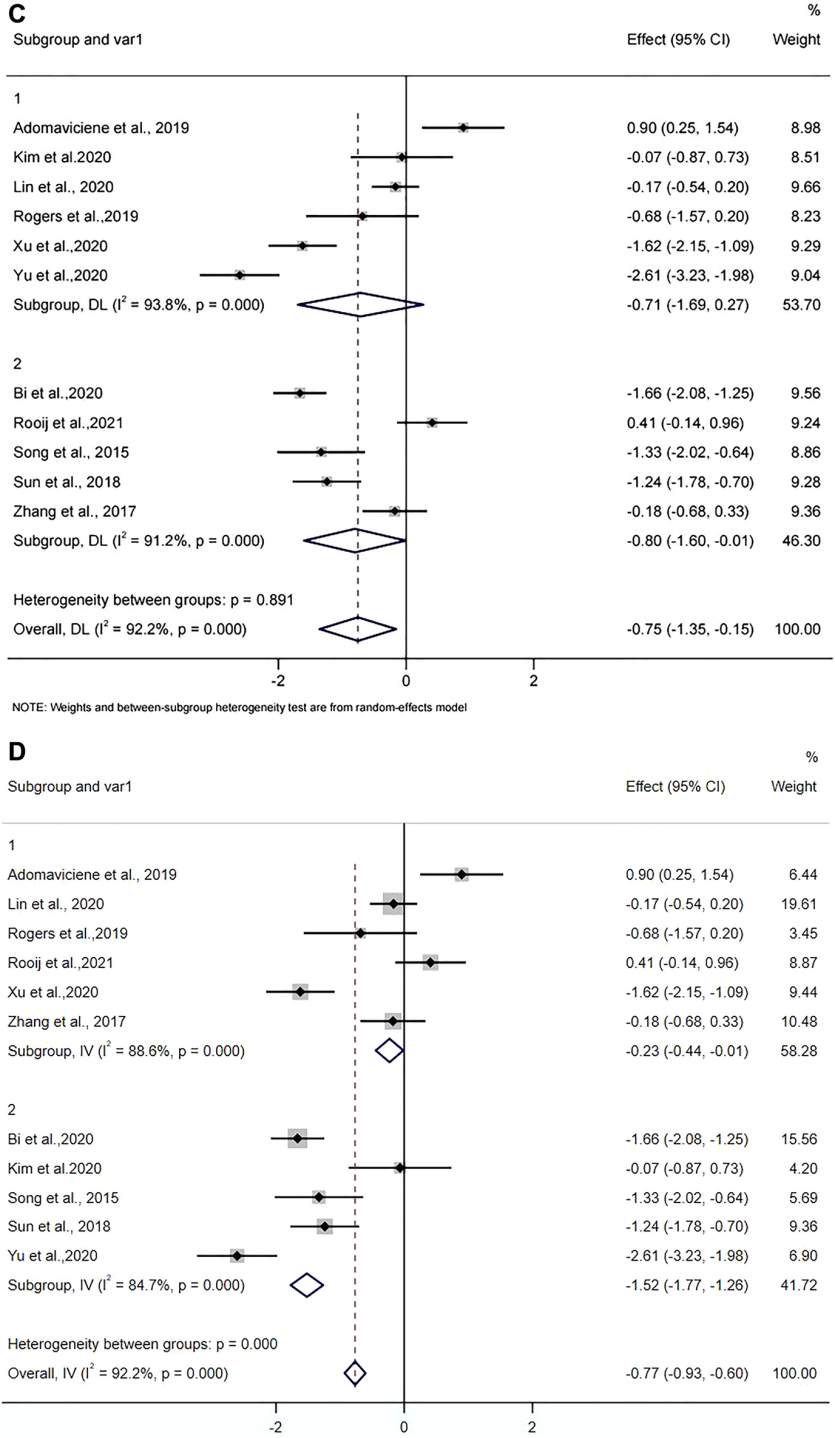
(**A**) Forest plot of the effect of VR on depression. (**B**) Forest plot of the effect of VR on depression in participants’ different mean age. (1 less than 60 years and 2 more than 60 years in figure). (**C**) Forest plot of the effect of VR on depression for different intervention duration (1 less than 6 weeks, and 2 more than 6 weeks). (**D**) Forest plot of the effect of VR on depression for different depression rating (1 mild depression, and 2 moderate depression).

**Figure 4:**
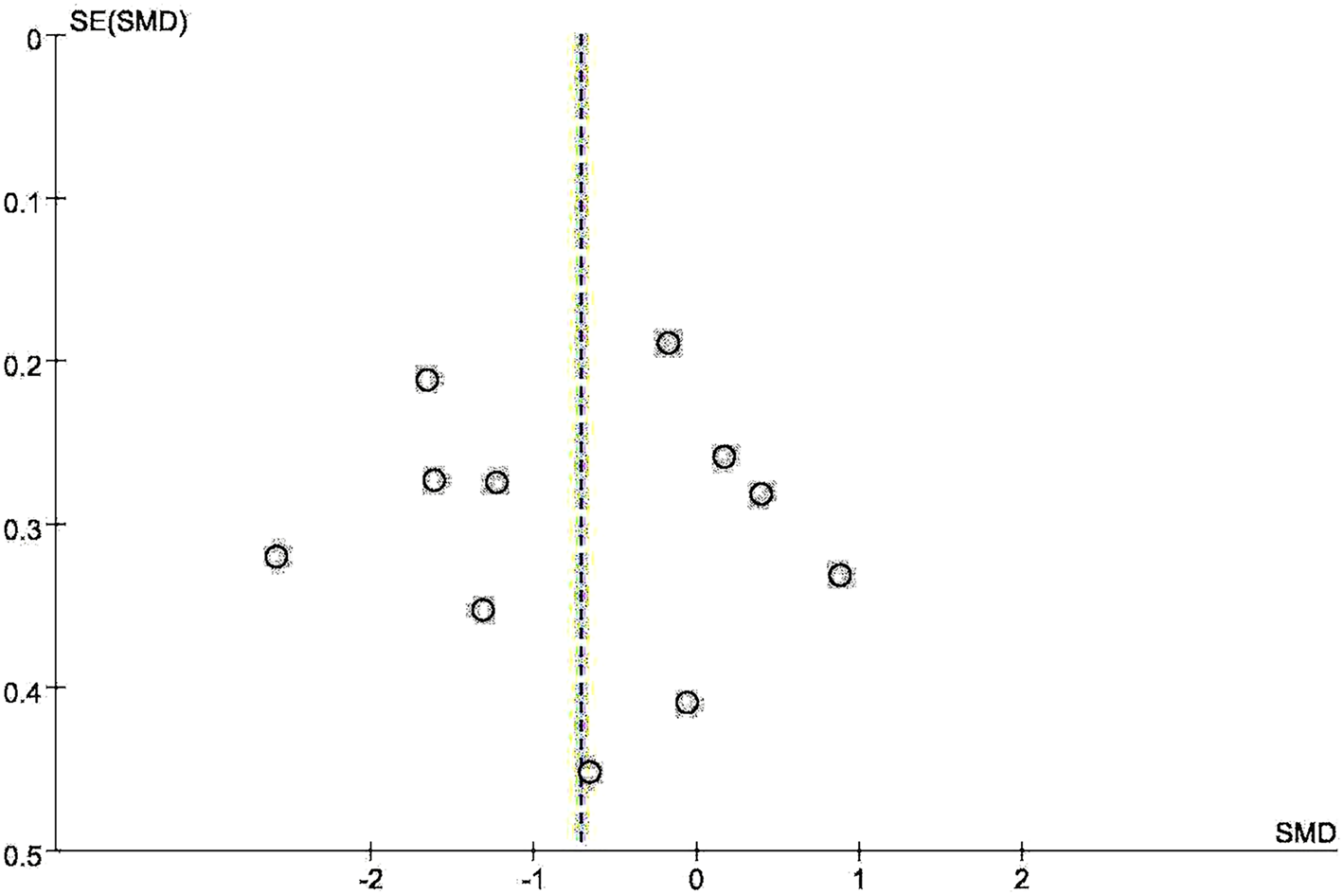
Funnel plot of the effect of VR on depression.

A total of 752 patients with stroke from 11 RCTs studies were included. The mean age of the four studies was under the age of 60, and the mean age of seven studies was over 60 years. The random effects model was used to pool the SMDs and corresponding 95% CIs. As shown in Fig. 3A, the results showed that depression scores in the VR group were significantly lower than that in the control group after the intervention (SMD =  − 0.75, 95% CI − 1.35, − 0.15). Significant heterogeneity was observed among the studies (*I*^2^ = 92.2%, *p* = 0.000). Egger test (*p* = 0.983) and Begg test (*p* = 0.755) (see Supplementary Appendix 3) and Funnel plot (in Fig. 4) showed no evidence for publication bias. The result of sensitivity analysis (see Supplementary Appendix 4) showed the heterogeneity did not decrease significantly and the pooled SMD varied from − 0.535 (− 1.111, − 0.042) (when excluding Yu et al.^43^) to − 0.881 (− 1.487, − 0.274) (when excluding Adomaviciene et al.^40^), which confirmed the robustness of the findings. The results indicated a high level of heterogeneity across the articles. Therefore, subgroup analysis and meta-regression analysis were used to further clarify the potential reasons for heterogeneity. Results of the meta-regression analysis showed that the heterogeneity could be explained by mean age (*P* = 0.029) (see Supplementary Appendix 5).

Subgroup analyses were performed based on WHO’s definition of the elderly (age < 60, age ≥ 60), the total intervention duration (< 6 weeks, ≥ 6 weeks), and depression rating (mild, moderate). The mean age of the four studies^42,43,46,47^ was under the age of 60, and the mean age of seven studies^5,31,39–41,44,45^ was over 60 years. As shown in Fig. 3B, the results indicated that depression scores in the VR group were reduced in participants with mean ages under 60 compared to control group (pooled effect size =  − 1.69, 95% CI − 2.29, − 1.10). Significant heterogeneity was observed among the studies (*I*^2^ = 75.1%, *p* = 0.007). However, depression scores are not reduced in participants with mean ages over 60 compared to controls (pooled effect size =  − 0.21, 95% CI − 0.88, 0.45). Significant heterogeneity was observed among the studies (*I*^2^ = 90.4%, *p* = 0.000).

For the duration of the intervention, five RCTs^39,44–47^ lasted longer than 6 × weeks, six RCTs^5,31,40–43^ were 1ess than 6 weeks. As shown in Fig. 3C, the results showed that depression scores in the VR group were statistically reduced for intervention session duration longer than 6 weeks compared to control group (pooled effect size =  − 0.80, 95% CI − 1.60, − 0.01). Significant heterogeneity was observed among the studies (*I*^2^ = 91.2%, *p* = 0.000). Depression scores in the VR group were decreased for intervention session duration 1ess than 6 weeks compared to control group (pooled effect size =  − 0.71, 95% CI − 1.69, 0.27). Significant heterogeneity was observed among the studies (*I*^2^ = 93.8%, *p* = 0.000).

Six of the studies^5,31,40,42,44,45^ involved people with mild depression, five^39,41,43,46,47^ included people with moderate depression and none with severe depression. As shown in Fig. 3D, the results indicated that depression scores in the VR group were significantly reduced in participants with moderate depression than control group (pooled effect size =  − 1.52, 95% CI − 1.77, − 1.26). Depression scores in the VR group were decreased in participants with mild depression compared to control group (pooled effect size = -0.23, 95% CI − 0.44, − 0.01). Significant heterogeneity was observed among the studies (*I*^2^ = 92.2%, *p* = 0.000).

## Discussion

Our meta-analysis systematically reviews currently available articles and included 11 studies with 752 patients to evaluate the effect of VR-based intervention on depression. The research results showed that depression score of VR-based intervention was significantly lower than that of the control group. The results are consistent with previous VR studies^14,30^ that have shown VR intervention to be beneficial in ameliorating depression.

The subgroup analysis divide’s the population by 60 years of mean age as a cutpoint, to focus on the impact of VR-based intervention on elderly stroke patients. The subgroup analysis suggests that VR intervention may have no effect on people over 60 years of age. For some older people, contacting VR technology has a challenging due to the visual and auditory changes^48^. This may be because older people have difficulty adapting to the new treatment regimen.

As to the total duration of the interventions, the subgroup analysis indicated that an intervention duration longer than 6 weeks was better than the intervention duration less than six weeks. In another meta-analysis, the results of meta-regression analysis showed that total intervention time had a significant effect on depression outcomes. It suggests that future VR interventions be conducted for at least 6 weeks, and the duration should be longer for greater efficiency against depressive outcomes.

Subgroup analysis of different depression ratings showed that VR-based intervention for patients with moderate depression was more effective than patients with mild depression. The result is consistent with previous literature that patients with higher baseline severity scores received more benefit from active treatments than from control conditions^49^.

If without social support, it has been confirmed that patients may have higher disability and medical burdens, and lower independence. Patients are at greater risk of developing PSD due to stroke-related defects after stroke^50–52^. The pathophysiological processes underlying PSD are multiple, complex, and incompletely understood^18,21,36^. Some of the main processes that may contribute to PSD include decreased levels of monoamines, abnormal neurotrophic response to stroke, increased inflammation with dysregulation of hypothalamicpituitary-adrenal (HPA) axis, and glutamate-mediated excitotoxicity^53,54^. Several potential mechanisms might explain the effect of VR-based intervention on depression, which may be through game-playing experiences and exercise training. In most VR interventions, rehabilitation programs are integrated into the game so that patients can have fun during the rehabilitation process. Evidence from neuropsychological researches shows the therapeutic value of game-based digital interventions in depression therapy, finding that positive game-playing experiences trigger the release of hormones such as endorphins and striatal dopamine^55,56^ that are responsible for feelings of pleasure and well-being^57,58^. In most VR interventions, rehabilitation programs also involve some level of exercise and being physically active. Rehabilitation exercise can help to rebuild brain structure can activate the function of related brain regions and promote adaptive behavioral changes, so that, it can promote the generation of positive emotions and improve the ability to cope with the depressive emotion.

VR as a therapeutic tool is rapidly popularized and widely used due to its novel training methods and ability to provide personalized rehabilitation training^59^. At present, various professional technology platforms and treatment schemes based on VR are also under active development and research^14^. The research results in recent decades showed that VR technology can be used to treat phobias, stress, and pain. But few have tried to develop VR anti-depressant interventions. Compared with traditional rehabilitation training, VR technology can precisely provide more individualized rehabilitation training programs according to the disease characteristics of different patients and can upload the training data on the internet in real-time. Through the data synchronization between different devices to improve the rehabilitation effect, the VR can also promote interaction between patients and the health system^6^.

Currently, no research results show which form of VR technical training or which treatment intensity has the best effect, and there is also a lack of guidelines on the VR development process. In future studies, the same interventions for patients having the same disease can be developed to eliminate high heterogeneity^14^. Few articles have evaluated or described feelings of immersion during treatment. The feelings of immersion should be used as an indicator for observation in future research, and the system needs to be to support improved outcomes to clarify how immersive and sophisticated. Recently, commercialized VR has developed rapidly, so, it is important to consider the cost, feasibility, and accessibility of VR equipment^60^. Few articles report the cost of VR rehabilitation training and the economic benefits of this type’s training have not been compared with the control group. In future studies, the cost of different types rehabilitation training should be used as an indicator for observation^14^. VR may also be used to alleviate depression symptoms and prevent depression in healthy people with some clinically related depression symptoms or sub-domain depression in the future. Based on evidence-based treatment techniques, consumer-targeted VR interventions have great potential to have an impact on the public’s psychological health.

There were some limitations should be noted in this study. It is different in the VR intervention forms, intervention period and frequency, and outcome evaluation scales adopted in the included studies. In addition, it is not quite the same that VR technology equipment used and the conventional physical rehabilitation mode implemented by the control group in each study. Above differences may be the reason for the high heterogeneity of results. Due to the particularity of VR technology interventions, the blind method is difficult for patients in the experimental group, which may lead to the deviation of subjective data reported by patients in the result evaluation.

## Conclusions and implications

The current meta-analysis indicates the VR-based intervention had a moderate effect on depression in stroke patients compared to control group. VR-based intervention session duration longer than 6 weeks was better than intervention session duration less than six weeks. For older adults with a mean age more than 60 years, depression scores of VR-based intervention on depression was not decreased than the control group. There was no evidence of potential publication bias based on the assessment of visual inspection to funnel plots Egger test and Begg test. Substantial heterogeneity between studies was observed, and meta-regression analysis showed that mean age might be the source of heterogeneity. This rehabilitation technology, suitable for promotion in family and community, has the advantages of saving manpower and low technical requirements. At the same time, clinical studies of different ages and different intervention duration also need further in-depth study.

## Supplementary Information

Supplementary Information 1.

Supplementary Information 2.

Supplementary Information 3.

Supplementary Information 4.

Supplementary Information 5.

Supplementary Information 6.

## Data Availability

All data generated or analyzed during the study are included in this published article and its Supplementary Information document.
