# Supplementary material for: Effects of virtual reality-based intervention on depression in stroke patients: a meta-analysis
Source: Sci Rep. 2023 Mar 16;13:4381. doi: 10.1038/s41598-023-31477-z (PMC10020160; doi:10.1038/s41598-023-31477-z)
Supplement: Supplementary file 1 — Supplementary Information 1. [file 41598_2023_31477_MOESM1_ESM.pdf]

Supplementary Appendix 1: Search strategy

Number	Search terms	Number	Search terms
1	Virtual reality	2	VR
3	virtual environment	4	1 or 2-3
5	depression	6	depressive disorder
7	depressive symptoms	8	5 or 6-7
9	stroke	10	Randomized controlled trial
11	RCT	12	Controlled clinical trial
13	Randomized	14	Randomly
15	Clinical trials	16	Random
17	9 or 10-15	18	4 and 8 and 9 and 17
