# Supplementary material for: Effects of virtual reality-based intervention on depression in stroke patients: a meta-analysis
Source: Sci Rep. 2023 Mar 16;13:4381. doi: 10.1038/s41598-023-31477-z (PMC10020160; doi:10.1038/s41598-023-31477-z)
Supplement: Supplementary file 2 — Supplementary Information 2. [file 41598_2023_31477_MOESM2_ESM.pdf]

Supplementary Appendix 2: Characteristics of Experimental group and Control group in the studies included the meta-analysis

Author, year	Experimental group	Control group	Dropouts and reasons	Depression rating
Adomaviciene et al., 2019	virtual reality Kinect-based system training+conventional rehabilitation training: virtual games+conventional rehabilitation training.	conventional rehabilitation training+armeo Spring robot-assisted training.	High blood pressure(n=6),Heart rate problems(n=4),Intolerance of physica l load(n=3),Fast fatigue(n=4),Painful shoulder syndrome(n= 3),<br>Fever(n=5),<br>Refusal to participate in the study(n= 3).	mild
Bi et al.,2020	VR psychological rehabilitation+conventional rehabilitation training：walking across zebra crossings, boating on the river, playing table tennis indoors and having a dialogue with the characters in the virtual scene.	conventional rehabilitation training.	not report.	moderate
Kim et l.2020	VR-based trunk-centered stabilization exercise.	trunk-centered stabilization exercise.	not report.	moderate
Lin et al., 2020	VR exercise+daily conventional occupational and physiotherapy.	conventional rehabilitation training(postural training, facilitation techniques, stretching exercise.	EG: n=0<br>CG: n=2(transferred intensive care unit).	mild
Rogers et al.,2019	VR exercise+daily conventional occupational and physiotherapy.	daily conventional occupational and physiotherapy.	not report.	mild
Rooij et al.,2021	VR gait training.	conventional treadmill training+functional gait exercises.	EG=0<br>CG=3(2recurrent stroke,1 reason unknown because of non-response).	mild

Song<br>et al., 2015	virtual reality games: 10-pin bowling, skiing, golf, ground walking, walking over obstacles, climbing stairs.	ergometer bicycle training.	not report.	moderate
Sun<br>et al., 2018	virtual reality games+conventional rehabilitation training+psychological intervention: Racing, big fish eat small fish, trajectory flight, trajectory tracking, picture matching, wipe the table, sports watch video, catch treasure, rebound ball, etc.	conventional rehabilitation training+psychological intervention.	not report.	moderate
Xu<br>et al.,2020	VR balance function training(track tracking training, mushroom picking training and walking training).	conventional rehabilitation training(range of motion training, balance function training, weight transfer training).	not report.	mild
Yu<br>et al.,2020	virtual reality games+stroke unit comprehensive treatment: parkour, matching cards, racing cars, squeezing toothpaste, holding a water glass, twisting towel.	stroke unit comprehensive treatment (internal medicine treatment, rehabilitation training;Comprehensive care, psychosocial and behavioral interventions, and post-rehabilitation career planning and vocational competency training)/conventional rehabilitation training.	not report.	moderate
Zhang<br>et al., 2017	virtual reality games+conventional rehabilitation training: Fruit cognition, life tool cognition, fruit cutting, ski event motion sensing game, speed skateboard.	conventional rehabilitation training: ①training for the maintenance of range of motion of lower limbs; ②muscle strength training; ③Bridge movement; ④training of gravity shift; ⑤sitting and standing balance training; ⑥sit-stand posture transfer training; ⑦General gait training.	not report.	mild
