# Supplementary material for: Effects of virtual reality-based intervention on depression in stroke patients: a meta-analysis
Source: Sci Rep. 2023 Mar 16;13:4381. doi: 10.1038/s41598-023-31477-z (PMC10020160; doi:10.1038/s41598-023-31477-z)

Supplementary Appendix 3: Egger's test and Begg's test

(1)Egger's test

▪ metabias_ES_seES, egger graph

Note: data input format theta se_theta assumed

Egger's test for small-study effects:

Regress standard normal deviate of intervention

▪

Number of studies=11 Root MSE=3.766

Std_Eff	Coef	Std. Err	t	P>| t |	[95% Conf. Intervl
Slope	-8181275	1.301327	-0.63	0.545	-3.761934 2.125679
Bias	0.1910248	4.685568	0.04	0.968	-10.40847 10.79052

Test of H0: no small-study effects P=0.968

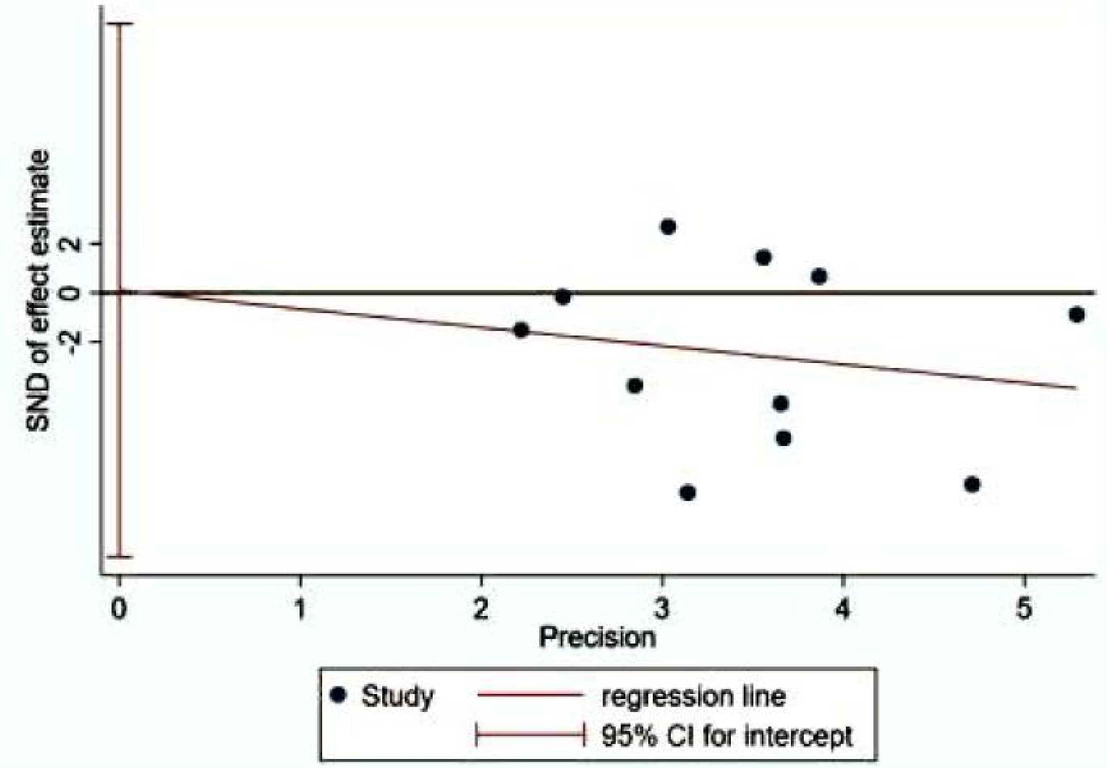

(2)Begg's test

▪ metabias_ES_seES, begg graph

Note: data input format theta se_theta assumed

Tests for Publication Bias

Begg's test

Adj. Kendall's Score (P-Q) = 5

Std. Dev. Of Score = 12.85

Number of Studies = 11

Z = 0.39

Pr> | z | = 0.697

Z = 0.31 (continuity corrected)

Pr> | z | = 0.697

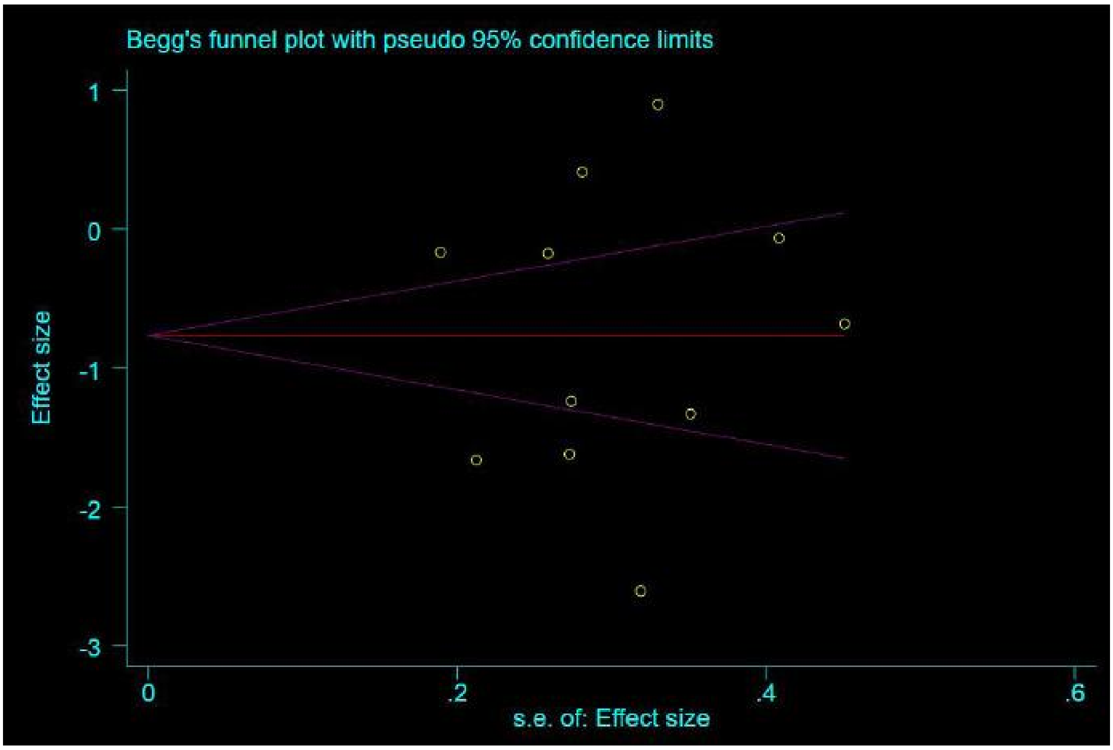

Supplement: Supplementary file 3 — Supplementary Information 3. [file 41598_2023_31477_MOESM3_ESM.pdf]
