# Supplementary material for: Effects of virtual reality-based intervention on depression in stroke patients: a meta-analysis
Source: Sci Rep. 2023 Mar 16;13:4381. doi: 10.1038/s41598-023-31477-z (PMC10020160; doi:10.1038/s41598-023-31477-z)

Supplementary Appendix 4: Sensitivity Analyses

- metaninf var2 var3 var4 var5 var6 var7, label(namevar = var1) random

Study omitted	Estimate	[95% Conf. Interval]	
Adomaviciene et al., 2019	-0.88075143	-1.4870754	-0.27442735
Bi et al., 2020	-0.62155944	-1.2676743	0.2455544
Kim et al., 2020	-0.78213763	-1.4387629	-0.12551236
Lin et al., 2020	-0.77909118	-1.4704815	-0.8770093
Rogers et al., 2019	-0.72420704	-1.3837827	-0.6463139
Rooij et al., 2021	-0.83614415	-1.4729548	-0.19933358
Song et al., 2015	-0.66129643	-1.3237352	0.00114234
Sun et al., 2018	-0.66776216	-1.3432703	0.00774592
Xu et al., 2020	-0.62892371	-1.284286	0.02643865
Yu et al., 2020	-0.53475112	-1.1112751	0.04177275
Zhang et al., 2017	-0.81321436	-1.4673965	-0.15903227
Combined	-0.72092549	-1.3379372	-0.10391379

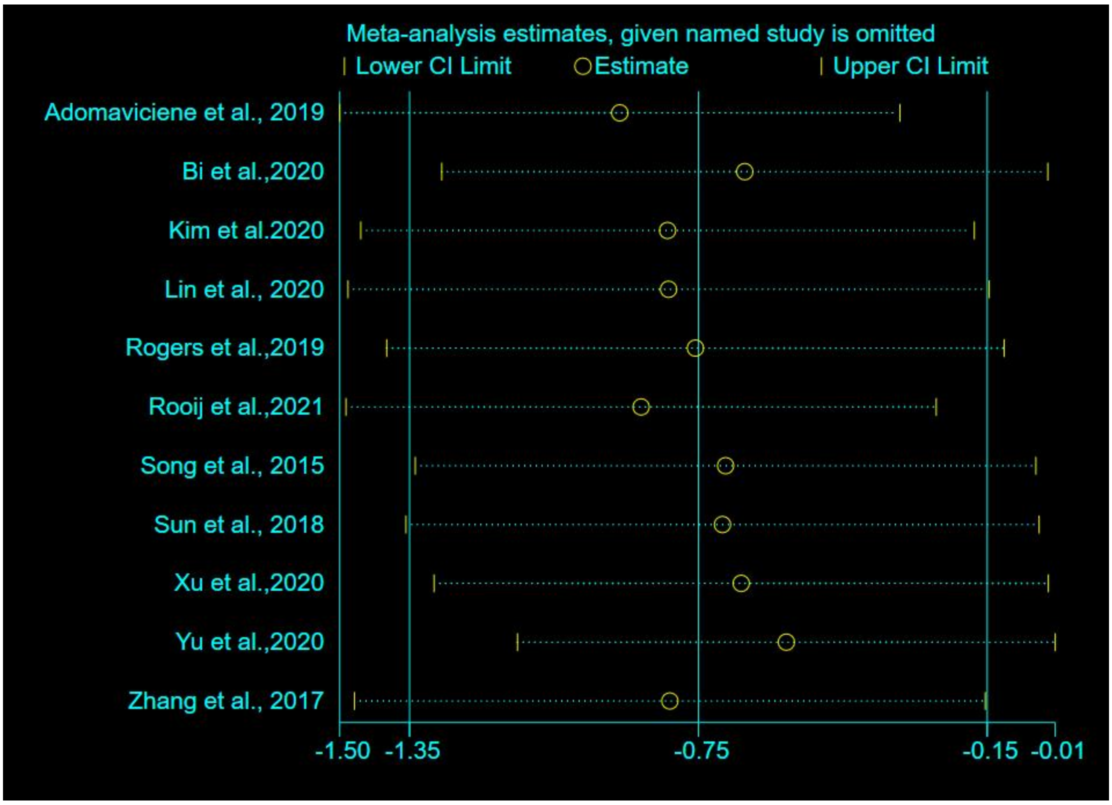

Supplement: Supplementary file 4 — Supplementary Information 4. [file 41598_2023_31477_MOESM4_ESM.pdf]
