# Supplementary material for: Effects of virtual reality-based intervention on depression in stroke patients: a meta-analysis
Source: Sci Rep. 2023 Mar 16;13:4381. doi: 10.1038/s41598-023-31477-z (PMC10020160; doi:10.1038/s41598-023-31477-z)
Supplement: Supplementary file 5 — Supplementary Information 5. [file 41598_2023_31477_MOESM5_ESM.pdf]

Supplementary Appendix 5: meta-regression

- metareg _ES age period country, wsse(_seES) bsest(reml)

Meta-regression	Number of obs	= 11
REML estimate of between-study variance	tau2	= 0.4077
% residual variation due to heterogeneity	I-squared_res	=82.70%
Proportion of between-study variance explained	Adj R-squared	=58.86%
Joint test for all covariates	Model F(3,7)	=4.80
With Knapp-Hartung modification	Prob>F	=0.0401

Covariate	Coef.	Std. Err.	t	P>|t|	95%CI
Age (year)	0.063769	0.0232666	2.74	0.029	-0.0087522, 0.1187857
Period	-0.1503881	0.1097952	-1.37	0.213	-0.4100125, 0.1092363
Country	0.2247958	0.1598416	1.41	0.202	-0.1531696, 0.6027611
_cons	-4.241404	1.549443	-2.74	0.029	-7.905253, -0.5775541
