# Supplementary material for: Effects of virtual reality-based intervention on depression in stroke patients: a meta-analysis
Source: Sci Rep. 2023 Mar 16;13:4381. doi: 10.1038/s41598-023-31477-z (PMC10020160; doi:10.1038/s41598-023-31477-z)
Supplement: Supplementary file 6 — Supplementary Information 6. [file 41598_2023_31477_MOESM6_ESM.pdf]

Supplementary Appendix 6: Raw data in manuscript

Study omitted	Experimental			Control		
	Mean	SD	Total	Mean	SD	Total
Adomaviciene et al., 2019	8.48	4.3	25	4.94	3.09	17
Bi et al., 2020	46.33	7.42	60	56.73	4.81	60
Kim et al., 2020	12.16	12.75	12	12.75	0.35	32
Lin et al., 2020	9.3	3.2	38	10	4.5	107
Rogers et al., 2019	24.5	6.6	10	30.6	10.6	11
Rooij et al., 2021	4.04	3.49	38	2.83	2.16	24
Song et al., 2015	14.1	2.4	20	17.5	2.7	30
Sun et al., 2018	8.7	2.8	31	12.3	3	33
Xu et al., 2020	10.17	2.01	36	14.26	2.95	36
Yu et al., 2020	11.25	2.12	40	16.24	1.64	34
Zhang et al., 2017	7.57	2.14	30	7.97	2.4	30
